# Quantitative evaluation of mesenchymal stromal cell immunomodulatory potency and cost-effectiveness of cytokine licensing for translational application

**DOI:** 10.1186/s12967-026-07947-z

**Published:** 2026-03-04

**Authors:** Dehua Liao, Jing Wen, Thomas Ritter, Jiemin Wang

**Affiliations:** 1 Department of Pharmacy, Hunan Cancer Hospital, The Affiliated Cancer Hospital of Xiangya School of Medicine, Central South University, Changsha, China; 2 Department of Pharmacy, The Third Hospital of Changsha, Changsha, China; 3 CÚRAM Centre for Research in Medical Devices, University of Galway, Galway, Ireland; 4 Regenerative Medicine Institute, School of Medicine, University of Galway, Galway, Ireland

**Keywords:** Mesenchymal stromal cells (MSCs), Immunomodulatory capacity, Cytokine licensing, Potency assay, Quantitative rating scale, Analytic Hierarchy Process (AHP), Principal Component Analysis (PCA), Cost-effectiveness analysis, Extracellular vesicles, Apoptotic bodies

## Abstract

**Background:**

Mesenchymal stromal cells (MSCs) possess strong immunomodulatory properties and are increasingly applied in inflammatory and immune-mediated diseases. Cytokine licensing, particularly with interferon-γ (IFN-γ), further enhances their therapeutic potential. However, standardized and quantitative approaches for evaluating MSC immunomodulatory capacity remain limited.

**Methods:**

We established a quantitative rating scale to assess the immunomodulatory capacity of MSCs and their extracellular vesicles. Using human bone marrow-derived MSC datasets, four weighting approaches, Analytic Hierarchy Process (AHP), Principal Component Analysis (PCA), the Entropy method, and the Independence method, were applied to determine the relative importance of indicators including T-cell suppression, regulatory T-cell induction, and key molecular markers. Cytokine licensing strategies were compared and paired with an economic feasibility assessment.

**Results:**

PCA and AHP performed best for indicator prioritization, with PCA yielding balanced weight distribution and AHP providing strong discriminative ability. Entropy and Independence methods emphasized variability and independence but showed weaker differentiation. IFN-γ was identified as the most effective licensing cytokine, while dual-licensing combinations such as IFN-γ/TGF-β1 achieved the highest overall immunomodulatory scores. Economic evaluation similarly favored IFN-γ–containing strategies, particularly in combination with TGF-β1.

**Conclusions:**

This study proposes an integrated framework combining immunomodulatory evaluation and economic feasibility to support standardized assessment of MSC function. The findings inform optimization of cytokine licensing strategies and may guide future clinical and economic decision-making for MSC-based therapies.

**Supplementary Information:**

The online version contains supplementary material available at 10.1186/s12967-026-07947-z.

## Introduction

Mesenchymal stromal cells (MSCs) have garnered significant attention in regenerative medicine due to their immunomodulatory properties and therapeutic potential in treating various inflammatory and immune-mediated diseases [1, 2]. The ability of MSCs to modulate immune responses is primarily mediated through their interactions with immune cells, including T cells [3–5], B cells [6, 7], and natural killer (NK) cells [8, 9]. These interactions involve a combination of cell-to-cell contact and the secretion of bioactive molecules, making MSCs a versatile tool in immunotherapy.

The immunomodulatory properties of MSCs are highly dynamic and can be significantly enhanced through preconditioning with pro-inflammatory cytokines such as interferon-gamma (IFN-γ) [10–15], tumor necrosis factor-alpha (TNF-α) [16–18], and interleukin-1 beta (IL-1β) [19–21]. These cytokines induce a robust immunomodulatory phenotype in MSCs, marked by the upregulation of key mediators like indoleamine 2,3-dioxygenase (IDO) [11, 14, 22] and programmed death-ligand 1 (PD-L1) [22–24]. In addition to the cytokines mentioned, our previous study demonstrated that TGF-β1-licensed MSCs can increase the frequency of regulatory T cells (Tregs) while reducing the secretion of pro-inflammatory biomolecules by macrophages [12, 13]. Furthermore, another study from our group confirmed that TGF-β1 enhances the immunomodulatory capacity of MSCs and modulates immune rejection in a murine model of corneal allografts [25].

Overall, recent research underscores the potential of cytokine-licensed MSCs to enhance therapeutic efficacy, particularly in the treatment of graft-versus-host disease [26, 27] and autoimmune disorders [28–30]. However, systematic approaches for quantifying the immunomodulatory capacity of MSCs remain underdeveloped and warrant further exploration.

Despite promising outcomes from cytokine-licensed MSCs in treating immune-related disorders, the development of standardized and comprehensive methods to assess their immunomodulatory potency remains a major challenge. The immunomodulatory potency of MSCs is a critical determinant of their therapeutic efficacy, yet standardized potency assays remain a challenge. The International Society for Cell & Gene Therapy (ISCT) emphasizes the need for robust methodologies to evaluate MSC function [31, 32]. Key assays include T-cell proliferation suppression tests [22, 33], which quantify MSC-mediated inhibition of activated T-cells, reflecting their direct immunoregulatory effects. Additionally, PD-L1 and IDO1 expression analyses provide mechanistic insights [22, 23], as these molecules are pivotal in MSC-driven immune evasion and tolerance induction. However, single-parameter assessments (e.g., T-cell suppression alone) often fail to capture the complexity of MSC mechanisms, as functional heterogeneity and donor variability influence outcomes [34]. Thus, a multidimensional potency evaluation framework, integrating functional and molecular metrics, is essential to ensure predictive and reproducible therapeutic potency. This approach aligns with ISCT guidelines [32], advocating for combinatorial assays to bridge the gap between in vitro characterization and in vivo efficacy.

In addition to their direct effects, MSCs release extracellular vesicles [35], including apoptotic bodies (ApoBDs) [13, 36–38], which play a crucial role in their immunomodulatory functions. These vesicles carry cytokines, nucleic acids, and proteins that can modulate immune responses in target cells. The impact of cytokine licensing on the cargo composition of these vesicles has been a focus of recent research [39, 40]. Our previous finding demonstrated that dual cytokine licensing with TGF-β1 and IFN-γ produces ApoBDs with enhanced immunosuppressive properties [13]. This finding suggests new possibilities for the development of MSC-derived extracellular vesicles as standalone therapeutic agents.

To evaluate the immunomodulatory capacity of MSCs (and their ApoBDs), it is critical to establish a standardized framework that captures the diverse mechanisms underlying their effects [32]. This study introduces a quantitative rating scale, incorporating multiple indicators of MSC function, such as T cell proliferation suppression, regulatory T cell induction, and expression levels of key immunomodulatory molecules. The indicators were categorized into two groups: (1) T cell responses after co-culture with MSCs and (2) intrinsic MSC-derived profiles. Four weighting methods—Analytic Hierarchy Process (AHP) [41], Principal Component Analysis (PCA) [42], Entropy [43], and Independence [44]—were applied to allocate weights to these indicators, providing distinct perspectives on their relative importance.

The therapeutic application of MSCs and their derivatives necessitates an evaluation of cost-effectiveness, particularly in the context of cytokine licensing. Cytokines such as IFN-γ and TGF-β1, while effective, contribute significantly to production costs. This study incorporates an incremental cost-effectiveness ratio (ICER) analysis to identify economically viable cytokine licensing strategies. By integrating ICER with the rating scale, this research aims to balance therapeutic efficacy with economic feasibility.

**Fig. 1:**
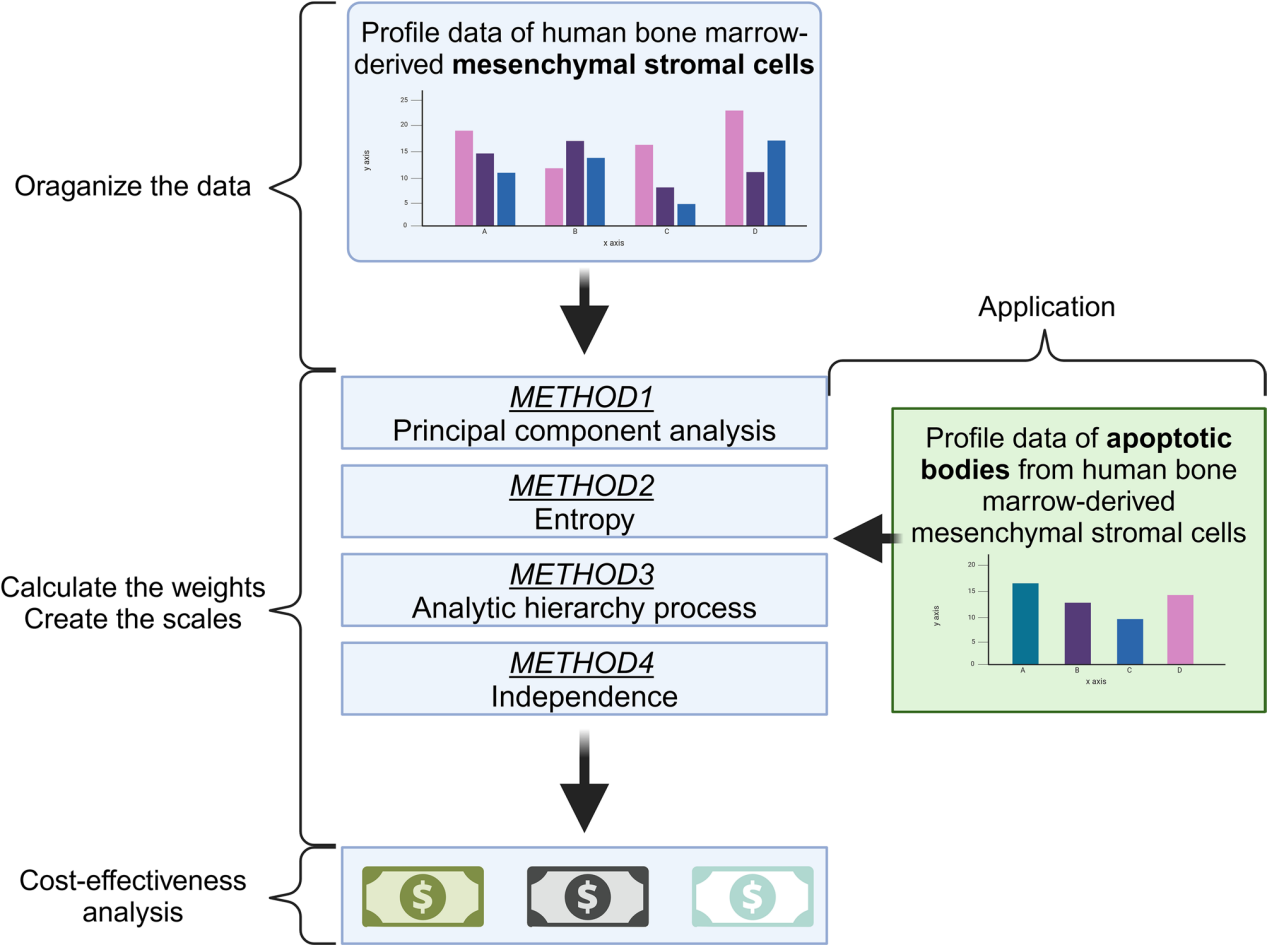
Workflow and conceptual framework of the study. The diagram illustrates the overall strategy, beginning with data collection from human bone marrow–derived mesenchymal stromal cells (MSCs), followed by weight calculation using four approaches (Principal Component Analysis, Entropy, Analytic Hierarchy Process, and Independence method), and subsequent application to apoptotic bodies (ApoBDs). Cost-effectiveness analysis was then conducted to evaluate immunomodulatory potential

In this study, we introduce a quantitative framework designed to standardize the evaluation of MSC immunomodulatory function, including both cells and their extracellular vesicles such as apoptotic bodies (ApoBDs) (Fig. 1). To achieve this, we establish a comprehensive rating scale and systematically compare four weighting strategies, AHP, PCA, Entropy, and Independence, to determine their suitability across different analytical and therapeutic contexts. We further investigate how cytokine licensing, particularly IFN-γ–based stimulation, modifies the immunomodulatory profiles of MSCs and their cargo. Finally, we integrate a cost-effectiveness assessment to examine the economic feasibility of various licensing strategies. Together, these components form a coherent framework that supports both scientific optimization and practical implementation of MSC-based therapies.

## Methods

### Data organization and preprocessing

This study utilized our previously published profiling data of human bone marrow-derived MSCs [12] and their ApoBDs [13]. The data is available in Additional File 2.

In brief, human bone marrow-derived MSCs were co-cultured with allogeneic peripheral blood mononuclear cells (PBMCs), and three biological replicates were conducted for this study. To address the issue of MSC heterogeneity, careful matching was implemented, ensuring balanced utilization of samples from multiple donors. Specifically, MSCs were donated by three volunteers, while PBMCs were donated by four individuals. The samples from these donors were strategically distributed across the biological replicates, minimizing variability due to donor-specific differences. Details on donor matching and experimental design can be found in the original publication [12, 13]. In other word, to minimize donor-specific heterogeneity, each biological replicate consisted of an independent MSC donor paired with an independent PBMC donor. Specifically, MSC donors A, B, and C were respectively co-cultured with PBMC donors 1, 2, and 3 (i.e., A→1, B→2, C→3).This one-to-one donor pairing ensured that each replicate reflected a unique MSC–PBMC combination, providing the most unbiased representation of donor variability.

The dataset encompassed various indicators of immunomodulatory capacity, including T cell proliferation, Treg induction, and the expression levels of key immunomodulatory molecules. To enhance data integrity and facilitate comprehensive analysis, these indicators were categorized into two groups (Table 1): (1) T cell responses to co-culture with MSCs and (2) intrinsic MSC-derived profiles, such as RNA and protein expression levels of immunomodulatory molecules.

Prior to analysis, raw data were preprocessed to handle missing values and ensure consistency across datasets. Z-score normalization was applied to each indicator to standardize the data and improve comparability across measurements.

As for the missing data described in the last paragraph, in Additional File 2, the raw dataset contained complete measurements for all indicators, and no missing values were present. The preprocessing script provided in Additional File 3 includes functions for handling missing values (e.g., na.rm = TRUE) because it is intended as a general-purpose template for reproducibility and learning. In our dataset, however, no imputation or data removal was required.

### Weighting methods

Four distinct methods were applied to calculate the weights for the immunomodulatory indicators:

1. PCA: PCA was utilized to identify principal components and assign weights according to each indicator’s contribution to these components. Indicators with high correlations (correlation coefficient > 0.8) were excluded to reduce multicollinearity and ensure the independence of the remaining indicators.
2. Entropy Method: The entropy method quantified the variability of each indicator, assigning higher weights to indicators with greater variability and information content. This approach emphasized data-driven weight allocation.
3. AHP: The AHP applied a hierarchical decision framework to prioritize indicators through expert judgment and pairwise comparisons. The pairwise comparison matrix was constructed based on two main criteria: (1) biological relevance, including the known mechanistic roles of indicators such as T-cell proliferation [13, 22, 33], IDO1 [22, 45], PD-L1 [22, 23], and Treg induction [46, 47]; and (2) evidence prevalence in the literature, particularly indicators repeatedly validated as potency markers in MSC immunomodulation studies. Indicators with well-established biological influence or strong literature support were assigned higher relative importance. The Consistency Ratio (CR) was calculated to ensure acceptable matrix consistency (CR < 0.1). The complete scoring matrix and AHP points are provided in Additional File 2.
4. Independence Method: To quantify indicator independence, we adopted a correlation-based independence weighting approach. For each indicator $i$, we calculated its independence score as the average anti-correlation with all other indicators, defined as:
  $$Independec{e_i} = {1 \over {n - 1}}\sum\limits_{j \ne i} {\left( {1 - \left| {{r_{ij}}} \right|} \right)} $$
  where ${r}_{ij}$ denotes the pairwise Pearson correlation coefficient between indicators $i$ and $j$. Indicators exhibiting lower absolute correlations (i.e., higher values of $1-\mid{r}_{ij}\mid$) were considered more independent and therefore received higher scores. The final independence weights were obtained by normalizing these scores across all indicators:
  $${\omega}_{i}=\frac{{Independence}_{i}}{{\sum}_{k=1}^{n}{Independence}_{k}}$$
  This method emphasizes non-redundant indicators and reduces the influence of variables that contribute overlapping information.

The detailed calculations and R-based code for this chapter are provided in the Additional File 1.

### Quantitative rating scale development

Using the weights derived from the four methods, a quantitative rating scale was developed to evaluate the immunomodulatory capacity of MSCs and their ApoBDs. Positive indicators (e.g., Tregs frequency, CD73 expression) were scaled to positively correlate with immunomodulatory capacity, while negative indicators (e.g., T cell proliferation frequencies) were scaled inversely.

$$\eqalign{& Immunomodulatory\,Capacity\,Score \cr& = \sum {\left( {Weigh{t_i}} \right) \times \left( {Standardized\,Indicato{r_i}} \right)} \cr& \times \left( {Directio{n_i}} \right) \cr} $$

${Weight}_{i}$: The weight assigned to indicator $i$.

${StandardizedIndicator}_{i}$: The Z-score of indicator $i$.

${Direction}_{i}$: A factor (+ 1 or -1) reflecting the relationship of the indicator to the target outcome.

### Validation of weighting methods

The weights calculated using the four methods were validated through consistency, differentiation validity, and correlation analyses. Cronbach’s Alpha and inter-item correlations were computed to assess consistency among the methods. ANOVA analysis was conducted to evaluate the ability of each method to distinguish between positive and negative indicators.

### Application to MSC and ApoBD data

The developed rating scale was applied to evaluate MSCs and their derived ApoBDs licensed with various cytokines, including IFN-γ, TGF-β1, TNF-α, and IL-1β. Immunomodulatory scores were calculated using each weighting method, and the results were compared to assess robustness and consistency.

### Cost-effectiveness analysis

The ICER was computed to evaluate the economic viability of cytokine licensing strategies. Cytokine prices were sourced from Additional File 2, and ICER values were calculated to assess the trade-off between cost and immunomodulatory enhancement. Groups with ICER values above zero were deemed cost-effective, indicating beneficial expenditure relative to naïve MSCs.

$$\eqalign{ICER & = {{\Delta Cost} \over {\Delta Score}} \cr& = {{Cos{t_{cytokine\left( s \right)}} - Cos{t_{naive\,MSCs}}} \over {Scor{e_{cytokine\left( s \right)}} - Scor{e_{naive\,MSCs}}}} \cr} $$

Numerator: Represents the additional cost incurred by using cytokine-licensed MSCs compared to naive MSCs.

Denominator: Represents the incremental improvement in the immunomodulatory capacity score achieved through cytokine licensing.

### Software and tools

All analyses were conducted using R (version 4.4.2). Key packages included “psych” for Cronbach’s Alpha, “FactoMineR” for PCA, and custom scripts for entropy and independence calculations. The processing scripts are provided in the Additional File 1. The diagrams were created using GraphPad (Prism 9).

## Results

### Arranging and normalizing the indicators

**Fig. 2:**
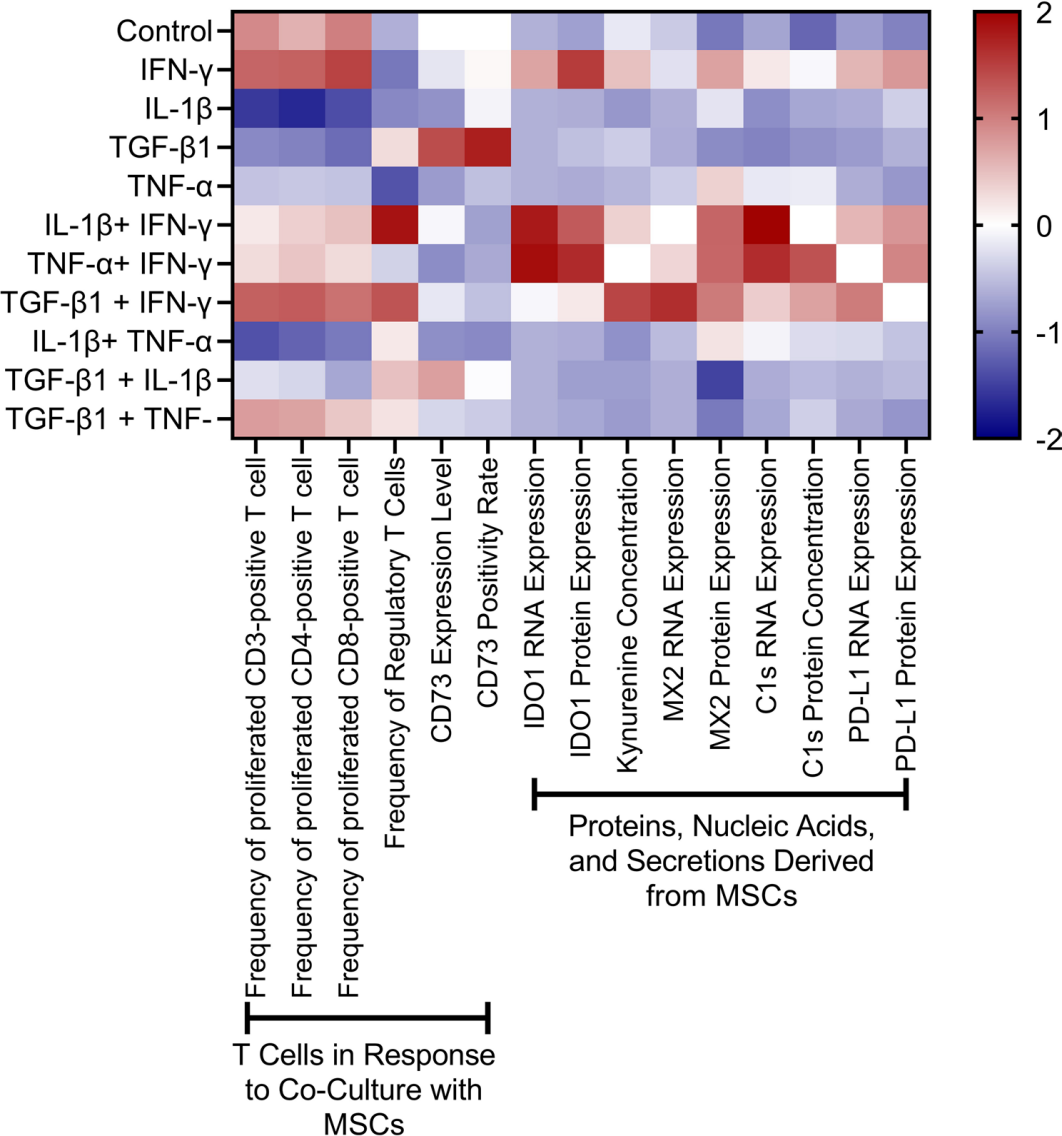
Standardized immunomodulatory profiles of mesenchymal stromal cells (MSCs) following stimulation with inflammatory cytokines. Data represent the mean values of three independent experiments. Indicators, including T cell responses and MSC-derived molecules, were normalized using Z-scores within each indicator. Red indicates higher expression or frequency, while blue indicates lower expression relative to the group mean

The first step is to normalize all the indicators, because the range of primary indicators are different. For example, as for T cell proliferation, the indicator ranges within 100% (The lower value indicates stronger immunomodulatory capacity for this indicator, whereas for the other indicators, higher values reflect stronger immunomodulatory capacity), while in terms of IDO1 expression, the indicator ranges within 10,000 depending on the median fluorescence intensity (MFI) of the protein. In the presented study, we used Z-score normalization to nomalize the value by each indicator. The normalized data are shown in Fig. 2. While the heatmap illustrates the varied immunomodulatory responses, it does not provide a comprehensive assessment of overall immunomodulatory capacity, underscoring the need for a quantitative rating scale.

### Preparing the rating scale

One of the primary aims of this study is to develop a comprehensive framework for quantifying the immunomodulatory capacity of MSCs. By assigning appropriate weights to various indicators of MSC function, this framework seeks to highlight their relative importance in assessing immunomodulatory potential. Indicators with higher weights are considered more influential in determining the overall capacity, underscoring their critical role in therapeutic evaluation and decision-making.

As shown in Table 1, the indicators, their directions, and corresponding weights are presented. Various methods, including AHP, Entropy, PCA, and the Independence method, were applied to assign weights. The R-based script used for weight calculation is provided in the Additional File 1.

**Table 1:** Weights assigned by various methods to quantify the immunomodulatory potency of mesenchymal stromal cells (MSCs)

Category	Indicators	Direction	Weight by			
			AHP	PCA	Entropy	Independence
T Cells in Response to Co-Culture with MSCs	Frequency of proliferated CD3-positive T cell	Negative	0.156	0.227	0.045	0.082
	Frequency of proliferated CD4-positive T cell	Negative	0.156	-	0.042	0.075
	Frequency of proliferated CD8-positive T cell	Negative	0.156	-	0.033	0.076
	Frequency of Regulatory T Cells	Positive	0.111	0.202	0.036	0.101
	CD73 Expression Level	Positive	0.096	0.170	0.071	0.095
	CD73 Positivity Rate	Positive	0.096	-	0.059	0.090
Proteins, Nucleic Acids, and Secretions Derived from MSCs	IDO1 RNA Expression	Positive	0.032	-	0.145	0.055
	IDO1 Protein Expression	Positive	0.032	-	0.098	0.054
	Kynurenine Concentration	Positive	0.032	0.193	0.076	0.056
	MX2 Expression	Positive	0.017	-	0.114	0.058
	MX2 Protein Expression	Positive	0.017	-	0.033	0.056
	C1s RNA Expression	Positive	0.017	0.207	0.060	0.049
	C1s Protein Concentration	Positive	0.017	-	0.040	0.049
	PD-L1 RNA Expression	Positive	0.032	-	0.088	0.051
	PD-L1 Protein Expression	Positive	0.032	-	0.059	0.052

Note: For the PCA method, certain indicators were excluded due to high multicollinearity, resulting in blank cells in the corresponding columns

The indicators were categorized into two groups: (1) T cells after co-culture with MSCs and (2) MSC-derived proteins, nucleic acids, and secretions. The former emphasizes alterations in T cells, while the latter focuses on the intrinsic characteristics of MSCs. In detail, the frequency of T cell proliferation is considered a gold standard for assessing the immunomodulatory capacity of MSCs [22, 31, 33, 48, 49]. Additionally, regulatory T cells (Tregs), a key T cell subtype with immunomodulatory effects [50], were included. The positivity and expression levels of CD73, a critical protein in immunomodulation [3, 51, 52], were also incorporated into the analysis. For the MSC-derived profile data, the study included RNA and protein levels of PD-L1 [23] and IDO1 [53], two well-documented immunomodulatory molecules. Furthermore, MX2 and C1s were included based on findings from our previous research [12], which identified these markers as upregulated in the gene profiles of highly immunomodulatory MSCs, as revealed by open-access MSC gene sequencing data [54–57]. This study also highlighted kynurenine (KYN), an amino acid secreted by IDO1, which was found to be elevated in conditions where IDO1 is upregulated [58, 59].

The direction of each indicator is an important consideration. Positive indicators signify that higher values correlate with stronger MSC immunomodulatory capacity (resulting in a higher overall score). In contrast, negative indicators imply that higher values are associated with weaker immunomodulatory capacity (resulting in a lower overall score). For example, the three types of T cell proliferation were classified as negative indicators because MSCs with stronger immunomodulatory potential suppress T cell proliferation, leading to lower indicator values.

Initially, following the AHP process, we estimated the importance of each indicator (Additional File 2) and subsequently calculated their weights. Briefly, the three types of T cell proliferation were assigned the highest importance, as they are considered the gold standard for assessing immunomodulatory capacity. The frequency of Tregs was deemed more important than CD73, as Tregs represent a more mature system and a distinct subtype. Next, the profile data of MSCs themselves were considered less critical than the alterations observed in T cells following MSC incubation. This prioritization reflects the fact that T cells are the recipient cells, and their responses are more indicative of MSC immunomodulatory potential. Among the indicators reflecting MSC properties, IDO1, KYN, and PD-L1 were given higher importance due to their frequent documentation in the literature [58, 60, 61]. In contrast, MX2 and C1s, being newly identified markers, were assigned relatively lower importance.

The weights calculated using AHP are based on a distinct set of evaluation criteria. The AHP estimation table is provided in Additional File 2. Specifically, the matrix dimension is *n* = 15, and the principal eigenvalue (λmax​) is 16.16488, slightly exceeding 15. This indicates a minor inconsistency in the judgment matrix. The Consistency Index (CI) quantifies the deviation from perfect consistency, with values closer to 0 indicating better consistency. While our results show a slight deviation in CI, it is not significant. The Consistency Ratio (CR) assesses whether the matrix’s consistency falls within an acceptable range. Generally, a CR value below 0.1 is considered acceptable. In our analysis, CR = 0.0558, which meets the consistency requirement and confirming that the judgment matrix’s consistency is within acceptable limits.

Secondly, the PCA method was applied to calculate the weights. PCA focuses on identifying the principal components and assigning higher weights to indicators that contribute more significantly to these components. However, due to the issue of multicollinearity among the selected indicators, adjustments were required. When indicators are highly correlated, their contributions to the principal component become similar, necessitating the removal of one from each correlated pair. The highly correlated indicators are listed in the Additional File 3.

After optimization, five indicators remained with acceptable levels of correlation: frequency of Tregs, KYN concentration, CD73 expression, frequency of proliferated CD3-positive T cells, and C1s RNA expression, as shown in the Table 1. The weights were distributed within a narrow range (approximately 0.17 to 0.23), indicating that the contributions of each variable to the total variance are relatively balanced, with no single variable holding a dominant position. Among them, the variable with the highest weight was frequency of proliferated CD3-positive T cells (22.73%), highlighting its relatively greater contribution to the variability of the data.

The biplot of Variable Contributions to Principal Components and the scree plot of Explained Variance by Principal Components are presented in the Additional File 3. The biplot illustrates the contributions of variables to the first two principal components (PC1 and PC2), which account for a significant portion of the variance (45.6% for PC1 and 26% for PC2). Key observations include the following:

- CD73 expression contributes strongly to PC1, as indicated by its large vector length in the positive direction.
- Treg and KYN exhibit moderate contributions, primarily along PC2.
- frequency of proliferated CD3-positive T cells is negatively correlated with PC1 and serves as a significant negative indicator.
- C1s RNA expression contributes to both PC1 and PC2 but has a relatively smaller impact compared to CD73 expression.

PC1 explains 45.6% of the variance, making it the most critical component, while PC2 accounts for 26%, capturing additional variability. Together, these two components explain approximately 71.6% of the total variance, justifying their use for weighting. This level of explained variance is acceptable for this analysis.

Lastly, the Entropy and Independence methods were applied to calculate the weights.

The Entropy method assigns weights based on the level of uncertainty or variability in the data. Specifically, it evaluates the degree of disorder or randomness (entropy) in the dataset. Indicators with higher variability or greater uncertainty contribute more to the system’s overall entropy and are typically assigned higher weights, as they carry more information content. Conversely, indicators with less variability or lower uncertainty are assigned lower weights, as they contribute less information to the analysis. According to the results, the weights calculated using the Entropy method ranged from 0.033 to 0.145, reflecting a higher degree of variability in certain indicators. Specifically, the gene fold changes, which exhibited a wide range from 1- to 1500-fold, were assigned relatively larger weights due to their substantial variability. In contrast, the frequency of proliferated T cells, which ranged from 10% to 30%, received smaller weights, even after normalization using Z-scores. This discrepancy highlights the greater influence of the variability in gene data compared to the more consistent proliferated T cell frequencies.

The Independence method is a weighting approach that emphasizes the degree of independence among indicators when assigning weights. It operates on the principle that indicators contributing unique and non-redundant information to the overall system should be prioritized over those with overlapping or redundant contributions. In our results, weights calculated by the Independence method ranged from 0.049 to 0.101, reflecting considerable variability in independence among the indicators. However, for indicators associated with Proteins, Nucleic Acids, and Secretions Derived from MSCs, the upregulated values showed a degree of correlation among themselves, indicating that they are not fully independent. As a result, their weights were relatively small, reflecting this lack of independence.

### Estimating the rating scale

In the previous chapter, four different rating scales for quantifying the immunomodulatory capacity of MSCs were generated using four distinct methods based on entirely different principles. Due to the issue of multicollinearity, several indicators were removed to ensure data independence and allow the PCA method to proceed for weight calculation.

#### Consistency

The weights derived from the other three methods were compared to evaluate consistency (Table 2).

**Table 2:** Consistency evaluation of the AHP, entropy, and independence methods

Raw Cronbach’s Alpha	Standardized Cronbach’s Alpha	Average Inter-Item Correlation	Signal-to-Noise Ratio
-0.244	-0.081	-0.025	-0.075

The results showed that both the raw Cronbach’s Alpha (-0.244) and the standardized Cronbach’s Alpha (-0.081) indicate very low consistency among the three methods. A negative Cronbach’s Alpha suggests that the data lack internal consistency, reflecting significant differences in weight allocation logic across methods. The Average Inter-Item Correlation, calculated as -0.025, highlights weak or negative relationships among the indicators, further emphasizing inconsistencies. Additionally, the Signal-to-Noise Ratio (SNR) of -0.075 indicates that noise within the data far outweighs the meaningful signal, further supporting the conclusion that there is a lack of alignment in the weight allocation logic across the methods.

#### Validity of the weight differentiation

**Table 3:** ANOVA analysis for validating weight differentiation

	Entropy	Independence	AHP
F-Statistic	2.819	1.495	28.89
p	0.117	0.243	0.000126

A between-groups ANOVA analysis was conducted to evaluate the validity of the weight differentiation process. The results, shown in Table 3, revealed that the AHP method had a statistically significant ability to distinguish between positive and negative indicators, with an F-Statistic of 28.89 and a p-Value of 0.000126. This indicates that the AHP-distributed weights effectively capture meaningful differences in indicator characteristics, enhancing its utility in weight allocation. In contrast, the Entropy and Independence methods displayed weaker differentiation of validity, as their weight distribution did not produce statistically significant distinctions between positive and negative indicators. This limitation underscores the challenges these methods face in effectively capturing the nuances of indicator variability.

#### Correlation

**Fig. 3:**
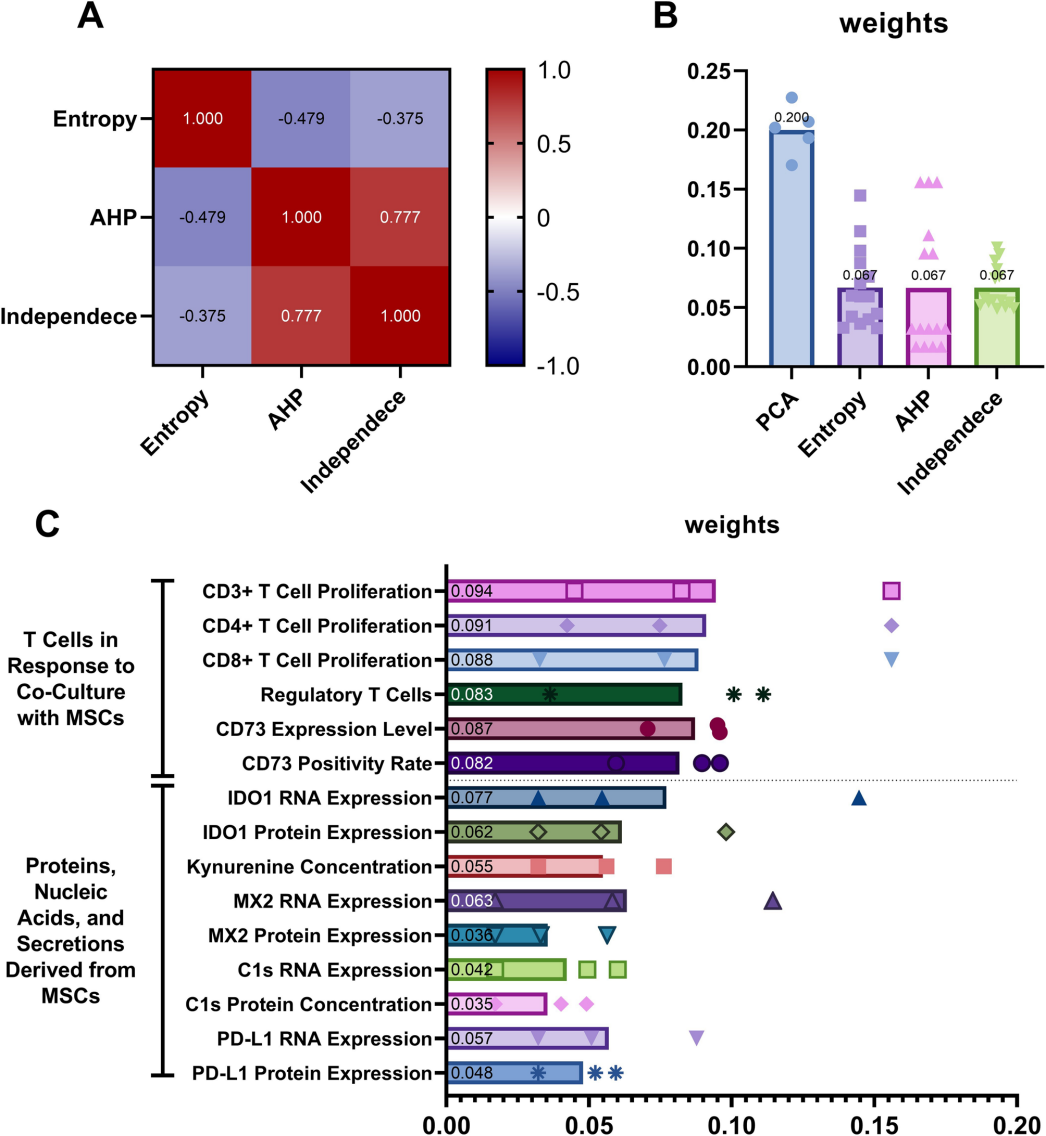
Evaluation of weight allocation methods. (**A**) Correlation matrix of weights derived from different methods. Mean values are shown. (**B**) Distribution of weights for each method, including Principal Component Analysis, Entropy method, Analytic Hierarchy Process, and Independence method. Mean values are shown. (**C**) Distribution of weights across individual indicators, with each dot representing a value derived from one method (Entropy method, Analytic Hierarchy Process, and Independence method). Data are based on three independent experiments. Mean values are shown

The weights allocated by the Entropy method differ significantly from the other two methods, with negative correlations predominating (Fig. 3A). This suggests that Entropy may be more focused on determining weights based on data variability, while being less sensitive to hierarchical or directional characteristics. In contrast, the weights allocated by AHP and Independence methods exhibit a much closer alignment, as indicated by their strong positive correlation. These methods show consistency in capturing data directionality and structural characteristics.

#### Weight distribution

The weight distributions across the four methods—PCA, Entropy, AHP, and Independence—exhibit distinct characteristics (Fig. 3B). Since only five indicators are involved, PCA assigns relatively high weights overall, ranging from 0.170 to 0.227. The range of PCA weights is relatively narrow, between 0.170 and 0.227, indicating a more balanced and uniform allocation across the five indicators due to the limited number of variables. Entropy produces lower and more concentrated weights, ranging from 0.036 to 0.076, as it relies on internal data variability. This approach ensures a balanced distribution, avoiding extreme values. AHP, on the other hand, displays the widest range of weights, from 0.017 to 0.156, emphasizing its ability to differentiate variable importance. Its hierarchical methodology prioritizes certain variables, resulting in a more variable distribution. Independence assigns weights in a moderate and consistent range of 0.049 to 0.100. By focusing on the relative independence of the indicators, it provides a distribution that is similar to Entropy in terms of uniformity but slightly higher in magnitude.

Indicators such as IDO1 RNA expression and MX2 RNA expression have higher Entropy weights but relatively low AHP and Independence weights (Fig. 3C), which may imply their importance stems from variability rather than decision-making frameworks. Secondly, Treg has a relatively high Independence weight and AHP weight, signifying it might be a stable predictor across frameworks. Negative-direction variables (T cell proliferation) generally have high AHP weights, showing their importance in decision-making hierarchies but relatively lower Entropy weights, suggesting lesser variability. Lastly, positive variables like CD73 expression level, PDL1 RNA expression, and IDO1 protein expression have balanced weights across all methods, highlighting them as consistent contributors.

#### Summary

AHP demonstrates more uneven weight distributions. Meanwhile, Entropy and Independence exhibit more balanced and lower weight allocations. These distinctions suggest that AHP are more suitable for prioritization tasks, while Entropy and Independence are better suited for scenarios requiring uniform weight distribution. The choice of method should depend on whether the focus is on indicator, hierarchical importance, or structural balance.

In summary, the four methods rely on distinct logical frameworks for weight allocation:

1. The PCA method is the simplest, involving only five indicators after removing highly correlated indicators. The PCA-specific weight allocation was deemed feasible, as it reduced redundancy and retained sufficient variability for analysis.
2. Among the remaining three methods, the AHP method demonstrated the best differentiation of validity, supported by its statistically significant results in the ANOVA analysis. Despite relying on subjective scoring of indicators, the AHP method produced weights that align well with the data’s underlying structure, making it a practical and reliable choice.
3. While the Entropy and Independence methods exhibited weaker differentiation of validity, they remain valuable for offering alternative perspectives on weight allocation. These methods are widely used in rating scale development [43, 44] due to their ability to handle complex datasets and provide unbiased results. The inconsistencies observed here highlight the importance of careful methodological selection based on the specific research context.

At present, AHP and PCA appear to be the more effective methods, offering better differentiation and feasibility in weight allocation. However, all four methods will continue to be applied in subsequent projects to calculate scores, allowing for a comprehensive understanding of indicator variability and cross-validation of results where necessary.

### Applying the data of MSCs and MSC-derived ApoBDs to the rating scale

#### MSC data

In the previous chapter, four types of rating scales were generated based on four distinct logical approaches. Among these methods, the weights allocated by PCA were the simplest and considered feasible. AHP was identified as the most effective method, while the Entropy and Independence methods were retained as they represent different perspectives. However, their effectiveness was limited compared to the AHP method in theory.

**Fig. 4:**
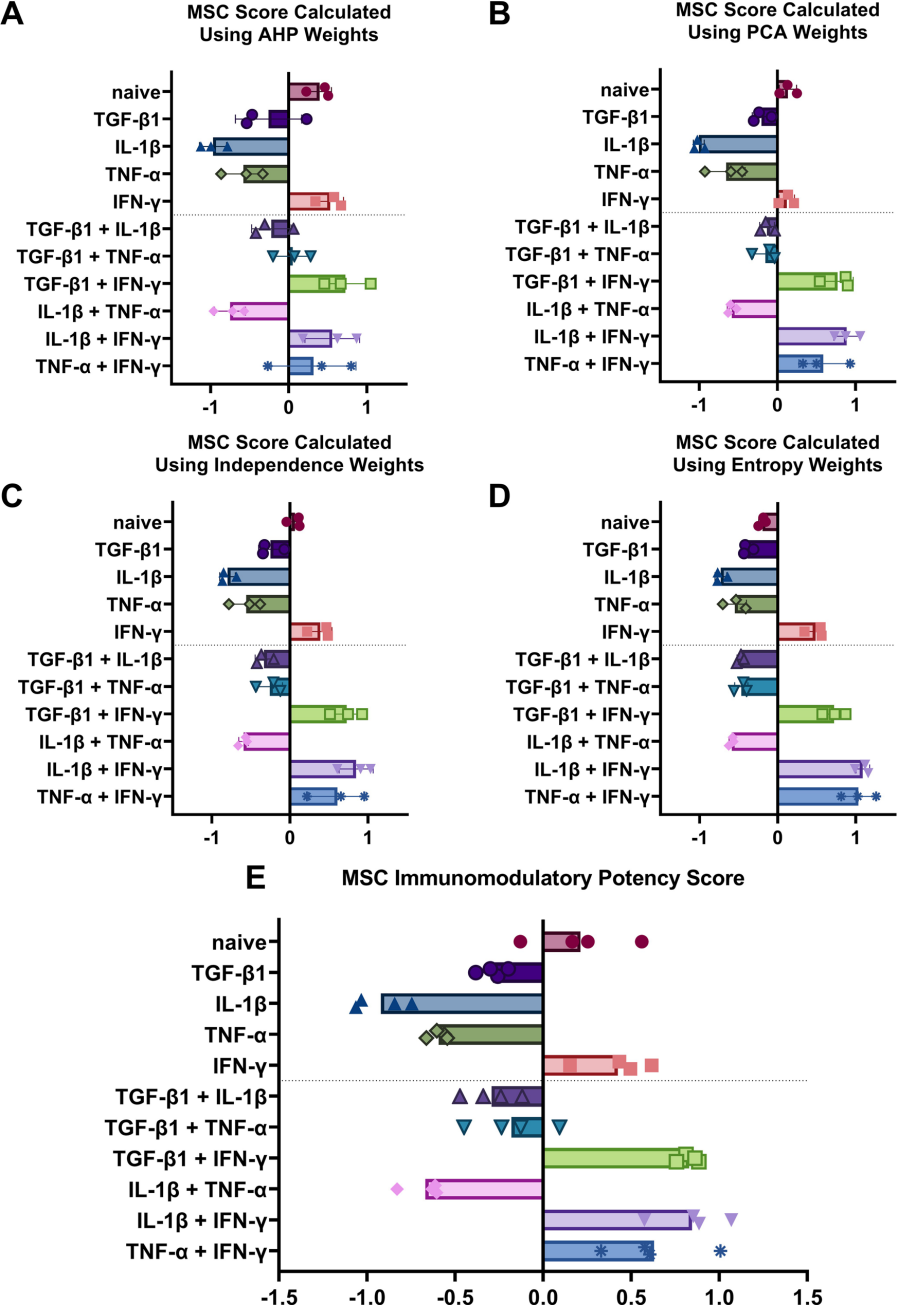
Immunomodulatory capacity scores of mesenchymal stromal cells (MSCs) using different weighting methods. Mean values are shown. (**A**) Analytic Hierarchy Process (AHP). (**B**) Principal Component Analysis (PCA). (**C**) Independence method. (**D**) Entropy method. (**A**–**D**) Each dot represents one biological replicate. (**E**) Integrated immunomodulatory potency score of MSCs across all four methods, with each dot representing the average score calculated per method

The results are shown in Fig. 4. As illustrated in Fig. 4A-D, remarkably consistent scores were observed across the rating scales, despite the completely different logical methodologies employed by the four rating scales. The immunomodulatory capacity scores for MSCs licensed with IFN-γ, including dual licensing combinations such as IFN-γ/TGF-β1, IFN-γ/TNF-α, and IFN-γ/IL-1β, were the highest. This finding aligns closely with our previous study [12], which concluded that IFN-γ is the most effective cytokine for inducing MSCs into an immunomodulatory phenotype. In contrast, single licensing with TNF-α, TGF-β1, or IL-1β was less effective in enhancing the immunomodulatory potency of MSCs.

As shown in Fig. 4E, each point represents the average score calculated using one rating scale from a specific weight allocation method. Among the results, the score of MSCs licensed with IFN-γ/TGF-β1 was the most stable, with all four rating scales producing approximately the same score (~ 0.9). While dual licensing with IFN-γ/TGF-β1, IFN-γ/TNF-α, and IFN-γ/IL-1β consistently achieved the highest scores, the scores for IFN-γ/TNF-α and IFN-γ/IL-1β showed a wider range (~ 0.3–1). This variability can be attributed to the fact that the combination of IFN-γ with TNF-α or IL-1β induces significant fold changes in genes such as MX2, C1s, PD-L1, and IDO1. These genes are assigned higher weights in the Entropy and Independence methods but receive lower weights in the AHP method, leading to relatively lower scores under the AHP approach.

The AHP method assigns higher weights to indicators related to T cell responses during co-culture with MSCs, which explains why naïve MSCs scored relatively high using this method. Naïve MSCs effectively modulate T cells but serve only as controls for gene and protein fold-change indicators, which are weighted less heavily in the AHP method.

The remaining cytokines or cytokine combinations achieved lower scores compared to IFN-γ, demonstrating that in our estimation system, IFN-γ is the most effective in inducing MSCs into an immunomodulatory phenotype.

Overall, these methods share the same goal—to evaluate the immunomodulatory capacity score. In our example, the Independence and Entropy methods also proved to be effective, though they appear less so when compared to the AHP method.

#### MSC-derived ApoBD data

Next, MSC-derived ApoBDs data from another previous study [13] was applied to the four rating scales. In this analysis, we focused exclusively on T cell data for the following reasons: (1) We place significant importance on T cells, as the alterations in recipient T cells are more representative of the effects of MSCs. (2) The rating scales have the potential to estimate the immunomodulatory capacity of other therapeutic agents, not limited to MSCs. (3) ApoBDs cannot synthesize proteins, and thus genetic changes within ApoBDs do not directly lead to phenotypic alterations in the vesicles themselves. Moreover, although apoptotic bodies can transfer functional regulatory RNAs, most notably microRNAs, to recipient cells, no study to date has provided evidence that mRNAs contained within ApoBDs can be translated into functional proteins. Current evidence indicates that the transductive capacity of ApoBDs is largely restricted to regulatory RNAs such as microRNAs, siRNAs, and lncRNAs [62–67]. Since the data available in this study were limited to messenger RNA, we therefore excluded RNA from the current framework.

**Fig. 5:**
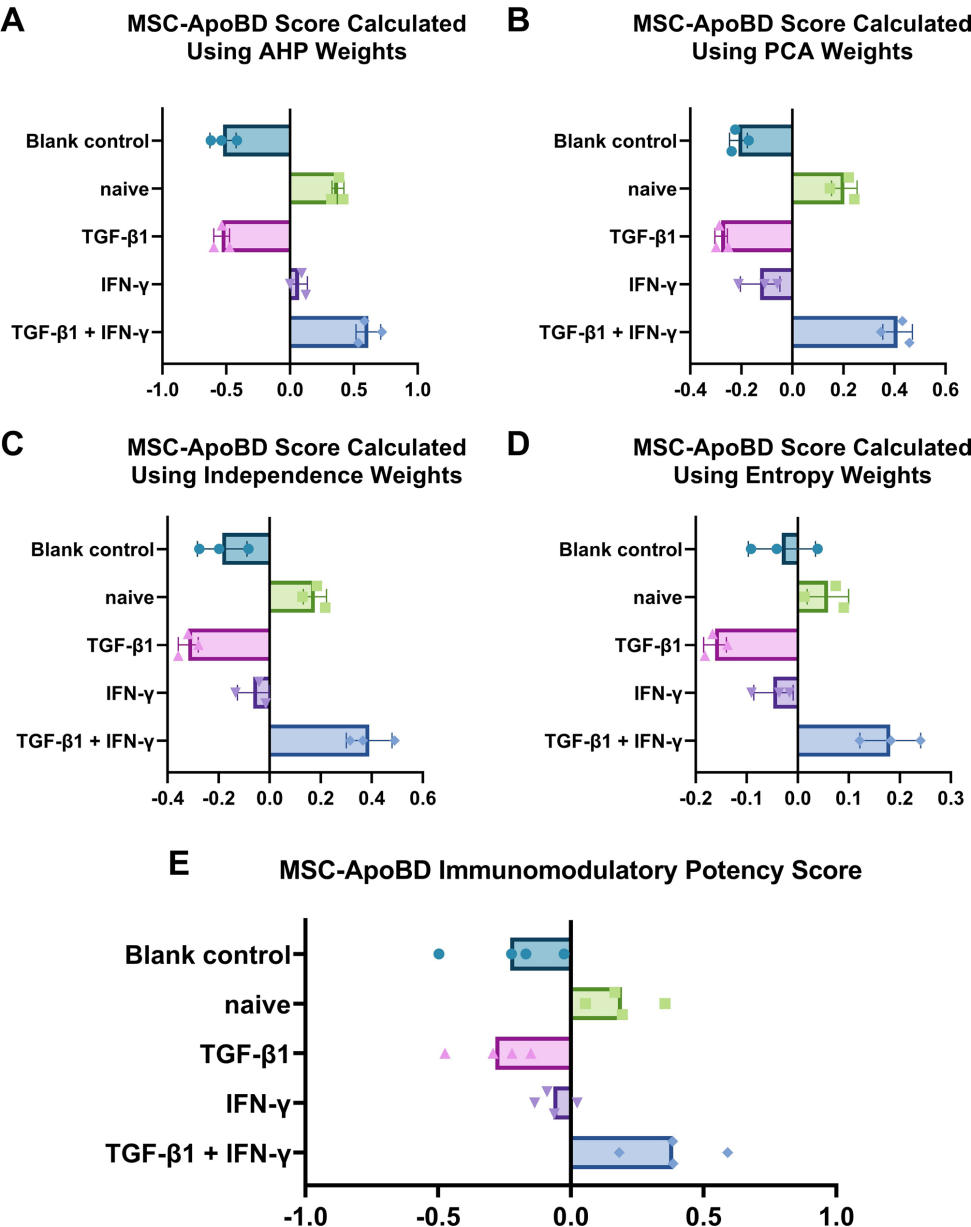
Immunomodulatory capacity scores of mesenchymal stromal cell-derived apoptotic bodies (MSC-ApoBDs) using different weighting methods. Mean values are shown. (**A**) Analytic Hierarchy Process (AHP). (**B**) Principal Component Analysis (PCA). (**C**) Independence method. (**D**) Entropy method. (**A**–**D**) Each dot represents one biological replicate. (**E**) Integrated immunomodulatory potency score of MSC-ApoBDs across all four methods, with each dot representing the average score calculated per method

As shown in Figs. 5A–D, the results were surprisingly consistent across all four rating scales. ApoBDs derived from naïve MSCs and IFN-γ/TGF-β1-licensed MSCs were found to be more effective than those from other groups. This finding is consistent with our earlier study [13], which concluded that ApoBDs derived from MSCs licensed with either TGF-β1 or IFN-γ alone exhibit limited immunomodulatory capacity. Consequently (Fig. 5E), these ApoBDs scored lower on all four rating scales. In contrast, ApoBDs derived from IFN-γ/TGF-β1-licensed MSCs consistently demonstrated the strongest immunomodulatory capacity across both the rating scales in this study and our previous findings [13].

### Cost-effectiveness analysis

**Fig. 6:**
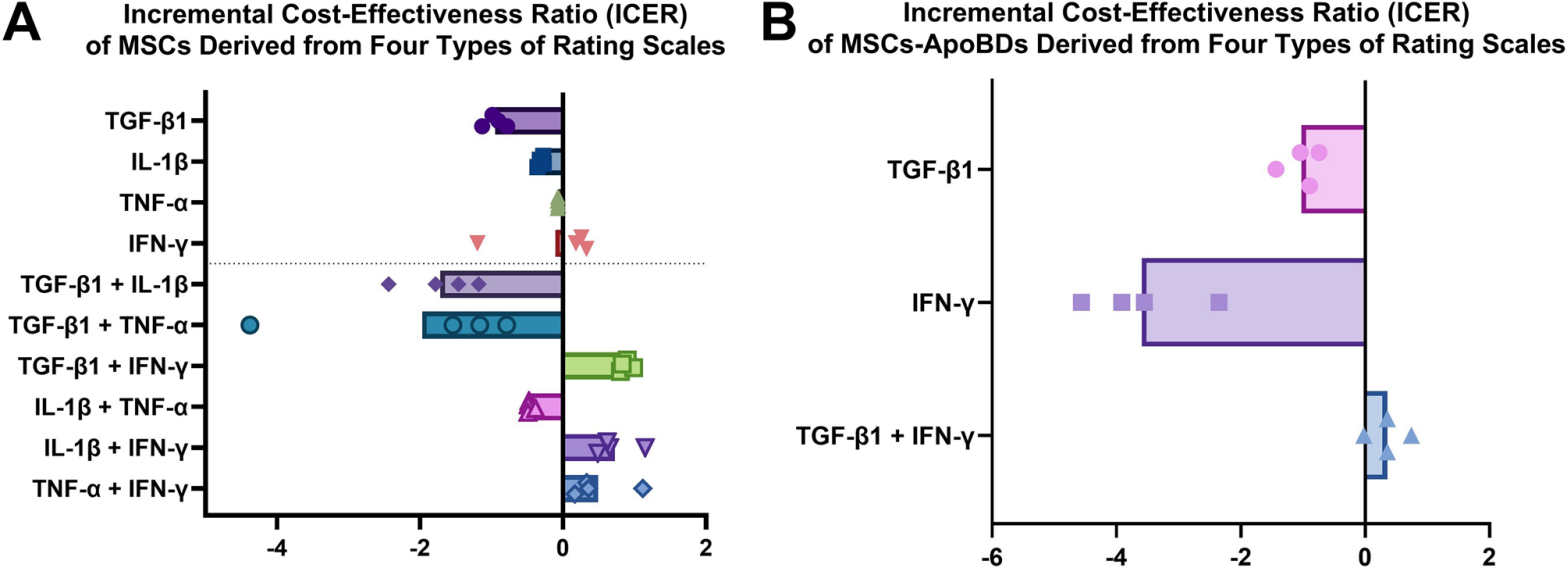
Incremental cost-effectiveness ratio (ICER) of mesenchymal stromal cells (MSCs) and their apoptotic bodies (ApoBDs). Mean values are shown. (**A**) ICER values of MSCs derived from four types of rating scales. (**B**) ICER values of MSC-ApoBDs derived from four types of rating scales. Each dot represents the average ICER calculated based on the weights generated by one method

Finally, the incremental cost-effectiveness ratio (ICER) was calculated to evaluate the cost-effectiveness of the selected cytokines and their combinations. The prices of the cytokines are provided in the Additional File 2. As shown in Fig. 6A, an ICER greater than zero indicates that the group has positive cost-effectiveness compared to naïve MSCs, meaning the expenditure results in a beneficial increase in immunomodulatory capacity. Conversely, an ICER below zero indicates that the expenditure fails to enhance immunomodulatory capacity.

The results (Fig. 6A) show that only groups containing IFN-γ demonstrated a positive effect, with the TGF-β1/IFN-γ dual-licensed model exhibiting the most stable positive cost-effectiveness. Among the four rating scale methods, the TGF-β1/IFN-γ group achieved the most consistent and highest cost-effectiveness-to-price ratio. Additionally, combinations of IFN-γ with TNF-α or IL-1β also showed good cost-effectiveness, but this was particularly evident with the Entropy method. The Entropy method assigned higher weights to RNA expression, an area where IFN-γ combined with TNF-α or IL-1β performed exceptionally well, resulting in higher scores. Furthermore, the other cytokine(s) clearly demonstrate a low effectiveness-to-cost ratio.

The ICER analysis was also applied to the ApoBD data (Fig. 6B). The findings revealed that the single use of IFN-γ or TGF-β1 resulted in financial loss, as their ICER values were below zero. While the TGF-β1/IFN-γ dual-licensed model demonstrated the highest cost-effectiveness among ApoBD groups, its cost-effectiveness remained limited, with an ICER value below one.

## Discussion

This study presents a quantitative rating scale for evaluating the immunomodulatory capacity of MSCs, leveraging four distinct weighting methods: AHP, PCA, Entropy, and Independence. The findings underline the unique strengths and limitations of these methods, providing valuable insights for their application in different research contexts.

The results demonstrate that PCA and AHP are more suitable for prioritization tasks, while Entropy and Independence excel in scenarios requiring uniform weight distribution. PCA, by focusing on variance explanation, provides a simplified framework that retains sufficient variability for analysis. AHP, on the other hand, excels in distinguishing between positive and negative indicators, as evidenced by the statistically significant results in ANOVA (F-Statistic = 28.89, *p* < 0.001). This highlights AHP’s ability to align weights with underlying data structure and logical hierarchies.

In contrast, Entropy and Independence methods emphasize different perspectives. The Entropy method assigns weights based on data variability, making it valuable in contexts where high variability is of interest. Independence, by prioritizing uncorrelated indicators, ensures minimal redundancy but may downplay correlated yet critical variables. Despite their weaker performance in differentiation validity, these methods provide alternative insights and are particularly useful in balancing indicator contributions.

The validation of the rating scale revealed consistency challenges among the methods. The negative Cronbach’s Alpha and weak inter-item correlations indicate significant differences in weight allocation logic. However, these inconsistencies offer complementary perspectives, enhancing the robustness of the overall analysis. AHP demonstrated the highest differentiation validity, supported by its alignment with data characteristics and hierarchical frameworks. PCA also performed well, particularly after adjustments for multicollinearity, where only five key indicators were retained.

The practical application of these methods was illustrated through the immunomodulatory capacity scores of MSCs under different licensing conditions. The findings corroborate the relatively strong effectiveness of IFN-γ licensing observed in this study, particularly in combination with TGF-β1, which consistently achieved the highest scores across all methods. This aligns with existing literature emphasizing the role of IFN-γ in inducing a potent immunomodulatory phenotype. Moreover, these results are consistent with the established understanding that IFN-γ licensing enhances MSC antigen-presenting plasticity [68, 69], IDO expression [70], and interaction with key immune subsets such as T cells [26]. The synergistic effect observed with TGF-β1 may reflect its capacity to further promote a regulatory phenotype, suggesting that dual-licensing strategies could more effectively prime MSCs for clinical scenarios characterized by heightened inflammation [12, 13, 25]. Integrating our scoring framework with these immunobiological insights highlights its potential utility for optimizing MSC preparation protocols and guiding the selection of more potent cell products for therapeutic use.

Furthermore, the cost-effectiveness analysis highlighted the economic viability of dual licensing strategies, particularly TGF-β1/IFN-γ. While single licensing with TNF-α or IL-1β showed limited cost-effectiveness, the Entropy method’s sensitivity to RNA expression variability identified potential advantages in certain scenarios.

Regarding the translational relevance of this framework, the quantitative evaluation system is particularly applicable to disease settings in which MSCs exert their therapeutic effects primarily through immunomodulation. Such indications include autoimmune diseases such as rheumatoid arthritis [71] and graft-versus-host disease (GvHD) [25], as well as inflammatory and tissue-repair conditions such as wound healing [72, 73]. In these disease contexts, cytokine-activated MSCs rely on immunoregulatory mechanisms, including T-cell suppression, macrophage polarization, and anti-inflammatory cytokine release, making the proposed framework directly relevant for comparing or optimizing licensing strategies across different clinical applications.

The major limitations of this study are twofold. First, it lacks in vivo validation. The primary aim here was to propose a conceptual framework for estimating MSC immunomodulatory capacity, whereas in vivo experiments will be conducted in future studies to provide further confirmation. Second, our study utilized MSCs from a single tissue source—bone marrow. Both MSCs and their derived ApoBDs were exclusively obtained from bone-marrow MSCs. Although the relatively homogeneous immunomodulatory capacity across MSC sources has been widely discussed, several studies have also reported source-dependent differences in MSC potency and functional profiles [74–76]. Therefore, it remains possible that adipose-, umbilical-cord-, or dental-pulp-derived MSCs may exhibit varied responses within this model. In subsequent work, MSCs from multiple tissue sources will be included to verify the generalizability and broader applicability of the framework. Third, our cost analysis focused primarily on the price of cytokine reagents. In reality, the major contributors to manufacturing cost, such as batch yield, quality-control testing, labor, facility operation, and regulatory compliance, often exceed reagent costs by a large margin. These clinical-scale parameters are difficult to accurately simulate under laboratory conditions. In future work, integrating large-scale manufacturing data and real-world cost structures will allow a more comprehensive evaluation of the economic impact of cytokine licensing.

In all, this study provides a comprehensive evaluation framework, future research could address several limitations:

1. Broader Application: The rating scale should be tested across diverse MSC sources and therapeutic contexts to ensure generalizability.
2. Advanced Consistency Metrics: Incorporating advanced consistency metrics, such as Cohen’s kappa or generalized agreement indices, could provide deeper insights into inter-method reliability.
3. Long-Term Cost-Effectiveness: Extending the cost-effectiveness analysis to account for long-term benefits, such as patient quality of life (QALYs) and reduced healthcare burden, would enhance its practical relevance.

## Conclusion

In summary, the choice of weighting methods should align with specific research objectives. In particular, AHP may be preferred for standard MSC potency release assays, especially those aligned with gold-standard T-cell suppression assays, whereas PCA is more suitable for exploratory analyses such as biomarker discovery. Entropy and Independence are more suitable when a more balanced weight distribution is desired. By providing a nuanced understanding of these methods, this study contributes to advancing the evaluation of MSC immunomodulatory capacity and informs the optimization of cytokine licensing strategies. Future efforts could refine these approaches, to improve their applicability across broader contexts and to better inform clinical and economic decision-making frameworks.

## Supplementary Information

Below is the link to the electronic supplementary material.

Supplementary Material 1

Supplementary Material 2

Supplementary Material 3

## Data Availability

The R scripts utilized in this study are included in Additional File 1, while the corresponding raw data are available in Additional File 2. For any further information or specific requests, please contact the corresponding author.

## Abbreviations

AHP: Analytic Hierarchy Process
ApoBD: Apoptotic body
ANOVA: Analysis of variance
ICER: Cost-effectiveness ratio
CI: Consistency Index
CR: Consistency Ratio
CRP: C-reactive protein
DEGs: Differentially expressed genes
EV: Extracellular vesicle
FBS: Fetal bovine serum
ICER: Incremental cost-effectiveness ratio
IDO: Indoleamine 2,3-dioxygenase
IFN-γ: Interferon-gamma
IL-1β: Interleukin-1 beta
MFI: Mean fluorescence intensity
MSC: Mesenchymal stromal cell
NTA: Nanoparticle tracking analysis
NO: nitric oxide
PBMC: Peripheral blood mononuclear cell
PCA: Principal Component Analysis
PD-L1: Programmed death-ligand 1
PGE2: Prostaglandin E2
PBS: Phosphate-buffered saline
Treg: Regulatory T cell
SNR: Signal-to-noise ratio
t-SNE: t-distributed stochastic neighbor embedding
TGF-β1: Transforming growth factor-beta 1
TNF-α: Tumor necrosis factor-alpha

## References

[1] Pang SHM, D’Rozario J, Mendonca S, Bhuvan T, Payne NL, Zheng D, et al. Mesenchymal stromal cell apoptosis is required for their therapeutic function. Nat Commun. 2021;12(1). 10.1038/s41467-021-26834-3 PMC8586224 34764248

[2] Galleu Y, Riffo-Vasquez C, Trento C, Lomas L, Dolcetti TS, Cheung, et al. Apoptosis in mesenchymal stromal cells induces in vivo recipient-mediated immunomodulation. Sci Transl Med. 2017;9(416). 10.1126/scitranslmed.aam7828 29141887

[3] Kerkela E, Laitinen A, Rabina J, Valkonen S, Takatalo M, Larjo A, et al. Adenosinergic Immunosuppression by Human Mesenchymal Stromal Cells Requires Co-Operation with T cells. Stem Cells. 2016;34(3):781–90. 26731338 10.1002/stem.2280

[4] Liu QL, Zheng HQ, Chen XY, Peng YW, Huang WJ, Li XB, et al. Human mesenchymal stromal cells enhance the immunomodulatory function of CD8 < SUP>+ CD28 < SUP>- regulatory T cells. Cell Mol Immunol. 2015;12(6):708–18. 25482073 10.1038/cmi.2014.118 PMC4716622

[5] Patel SR, Copland IB, Garcia MA, Metz R, Galipeau J. Human mesenchymal stromal cells suppress T-cell proliferation independent of heme oxygenase-1. Cytotherapy. 2015;17(4):382–91. 25595329 10.1016/j.jcyt.2014.11.010

[6] Yoshioka S, Miura Y, Yao H, Satake S, Hayashi Y, Tamura A, et al. CCAAT/Enhancer-Binding Protein β Expressed by Bone Marrow Mesenchymal Stromal Cells Regulates Early B-Cell Lymphopoiesis. Stem Cells. 2014;32(3):730–40. 24115241 10.1002/stem.1555

[7] Barrio L, Cuevas VD, Menta R, Mancheño-Corvo P, delaRosa O, Dalemans W, et al. Human adipose tissue-derived mesenchymal stromal cells promote B-cell motility and chemoattraction. Cytotherapy. 2014;16(12):1692–9. 25240680 10.1016/j.jcyt.2014.07.012

[8] Abdelrazik H, Spaggiari GM, Chiossone L, Moretta L. Mesenchymal stem cells expanded in human platelet lysate display a decreased inhibitory capacity on T- and NK-cell proliferation and function. Eur J Immunol. 2011;41(11):3281–90. 21874650 10.1002/eji.201141542

[9] Hu CHD, Kosaka Y, Marcus P, Rashedi I. Differential Immunomodulatory Effects of Human Bone Marrow-Derived Mesenchymal Stromal Cells on Natural Killer Cells. Stem Cells Dev. 2019;28(14):933–43. 31122145 10.1089/scd.2019.0059

[10] Guess AJ, Daneault B, Wang RZ, Bradbury H, La Perle KMD, Fitch J, et al. Safety Profile of Good Manufacturing Practice Manufactured Interferon gamma-Primed Mesenchymal Stem/Stromal Cells for Clinical Trials. Stem Cells Translational Med. 2017;6(10):1868–79. 10.1002/sctm.16-0485 PMC6430053 28887912

[11] Kwee BJ, Lam J, Akue A, KuKuruga MA, Zhang KY, Gu L, et al. Functional heterogeneity of IFN-γ-licensed mesenchymal stromal cell immunosuppressive capacity on biomaterials. Proc Natl Acad Sci USA. 2021;118(35). 10.1073/pnas.2105972118 PMC8536328 34446555

[12] Wang J, Zhou Y, Donohoe E, Canning A, Moosavizadeh S, Ryan AE, et al. Immunomodulatory potential of cytokine-licensed human bone marrow-derived mesenchymal stromal cells correlates with potency marker expression profile. Stem cells (Dayton, Ohio). 2024. 10.1093/stmcls/sxae053 PMC11630899 39208292

[13] Jiemin Wang E, Donohoe A, Canning S, Moosavizadeh F, Buckley MA, Brennan, et al. Immunomodulatory function of licensed human bone marrow mesenchymal stromal cell-derived apoptotic bodies. Int Immunopharmacol. 2023;125(Pt A):111096. 37871378 10.1016/j.intimp.2023.111096

[14] Chinnadurai R, Copland IB, Patel SR, Galipeau J. IDO-Independent Suppression of T Cell Effector Function by IFN-γ-Licensed Human Mesenchymal Stromal Cells. J Immunol. 2014;192(4):1491–501. 24403533 10.4049/jimmunol.1301828

[15] Chinnadurai R, Copland IB, Garcia MA, Petersen CT, Lewis CN, Waller EK, et al. Cryopreserved Mesenchymal Stromal Cells Are Susceptible to T-Cell Mediated Apoptosis Which Is Partly Rescued by IFNγ Licensing. Stem Cells. 2016;34(9):2429–42. 27299362 10.1002/stem.2415 PMC5016228

[16] Murphy N, Treacy O, Lynch K, Morcos M, Lohan P, Howard L, et al. TNF-α/IL-1β-licensed mesenchymal stromal cells promote corneal allograft survival via myeloid cell-mediated induction of Foxp3 + regulatory T cells in the lung. FASEB J. 2019;33(8):9404–21. 31108041 10.1096/fj.201900047R

[17] Boland L, Burand AJ, Brown AJ, Boyt D, Lira VA, Ankrum JA. IFN-γ and TNF-α Pre-licensing Protects Mesenchymal Stromal Cells from the Pro-inflammatory Effects of Palmitate. Mol Ther. 2018;26(3):860–73. 29352647 10.1016/j.ymthe.2017.12.013 PMC5910660

[18] Lu ZF, Chen YJ, Dunstan C, Roohani-Esfahani S, Zreiqat H. Priming Adipose Stem Cells with Tumor Necrosis Factor-Alpha Preconditioning Potentiates Their Exosome Efficacy for Bone Regeneration. Tissue Eng Part A. 2017;23(21–22):1212–20. 28346798 10.1089/ten.tea.2016.0548

[19] Yu Y, Yo SM, Park HH, Baek SY, Kim YJ, Lee S, et al. Preconditioning with interleukin-1 beta and interferon-gamma enhances the efficacy of human umbilical cord blood-derived mesenchymal stem cells-based therapy via enhancing prostaglandin E2 secretion and indoleamine 2,3-dioxygenase activity in dextran sulfate sodium-induced colitis. J Tissue Eng Regen Med. 2019;30:1792–804. 10.1002/term.2930 31293088

[20] Liu HC, Zhu XN, Cao XH, Chi A, Dai J, Wang ZQ, et al. IL-1 beta-primed mesenchymal stromal cells exert enhanced therapeutic effects to alleviate Chronic Prostatitis/Chronic Pelvic Pain Syndrome through systemic immunity. Stem Cell Res Ther. 2021;12(1). 10.1186/s13287-021-02579-0 PMC8466748 34563249

[21] Magne B, Dedier M, Nivet M, Coulomb B, Banzet S, Lataillade JJ, et al. IL-1β-Primed Mesenchymal Stromal Cells Improve Epidermal Substitute Engraftment and Wound Healing via Matrix Metalloproteinases and Transforming Growth Factor-β1. J Invest DERMATOLOGY. 2020;140(3):688–. 10.1016/j.jid.2019.07.721 31513805

[22] Guan Q, Li Y, Shpiruk T, Bhagwat S. Wall. Inducible indoleamine 2,3-dioxygenase 1 and programmed death ligand 1 expression as the potency marker for mesenchymal stromal cells. Cytotherapy. 2018;20(5):639–49. 29548707 10.1016/j.jcyt.2018.02.003

[23] Strauch V, Saul D, Berisha M, Mackensen A, Mougiakakos D, Jitschin R. N-glycosylation controls inflammatory licensing-triggered PD-L1 upregulation in human mesenchymal stromal cells. Stem Cells. 2020;38(8):986–93. 32346937 10.1002/stem.3190

[24] Grinnemo KH, Löfling M, Nathanson L, Baumgartner R, Ketelhuth DFJ, Beljanski V, et al. Immunomodulatory effects of interferon-γ on human fetal cardiac mesenchymal stromal cells. Stem Cell Res Ther. 2019;10(1). 10.1186/s13287-019-1489-1 PMC6894330 31801632

[25] Lynch K, Treacy O, Chen X, Murphy N, Lohan P, Islam MN, et al. TGF-beta1-Licensed Murine MSCs Show Superior Therapeutic Efficacy in Modulating Corneal Allograft Immune Rejection In Vivo. Mol Ther. 2020;28(9):2023–43. 32531237 10.1016/j.ymthe.2020.05.023 PMC7474271

[26] Polchert D, Sobinsky J, Douglas GW, Kidd M, Moadsiri A, Reina E, et al. IFN-γ activation of mesenchymal stem cells for treatment and prevention of graft versus host disease. Eur J Immunol. 2008;38(6):1745–55. 18493986 10.1002/eji.200738129 PMC3021120

[27] Wang YJ, Xi YF, Han F, Liu Y, Li N, Ren ZL, et al. Vascularized composite allograft rejection is delayed by infusion of IFN-γ-conditioned BMSCs through upregulating PD-L1. Cell Tissue Res. 2019;376(2):211–20. 30613905 10.1007/s00441-018-2967-y

[28] Tsiapalis D, Floudas A, Tertel T, Boerger V, Giebel B, Veale DJ, et al. Therapeutic Effects of Mesenchymal/Stromal Stem Cells and Their Derived Extracellular Vesicles in Rheumatoid Arthritis. STEM CELLS TRANSLATIONAL Med. 2023;12(12):849–62. 10.1093/stcltm/szad065 PMC10726408 37934808

[29] Yang Y, He X, Zhao RS, Guo W, Zhu M, Xing W, et al. Serum IFN-γ levels predict the therapeutic effect of mesenchymal stem cell transplantation in active rheumatoid arthritis. J Transl Med. 2018;16. 10.1186/s12967-018-1541-4 PMC6003078 29903026

[30] He X, Yang Y, Yao MW, Yang L, Ao LQ. Combination of human umbilical cord mesenchymal stem (stromal) cell transplantation with IFN-γ treatment synergistically improves the clinical outcomes of patients with rheumatoid arthritis. Ann Rheum Dis. 2020;79(10):1298–304. 32561603 10.1136/annrheumdis-2020-217798

[31] De Wolf C, Van De Bovenkamp M, Hoefnagel M. Regulatory perspective on in vitro potency assays for human mesenchymal stromal cells used in immunotherapy. Cytotherapy. 2017;19(7):784–97. 28457740 10.1016/j.jcyt.2017.03.076

[32] Galipeau J, Krampera M, Barrett J, Dazzi F, Deans RJ, Debruijn J, et al. International Society for Cellular Therapy perspective on immune functional assays for mesenchymal stromal cells as potency release criterion for advanced phase clinical trials. Cytotherapy. 2016;18(2):151–9. 26724220 10.1016/j.jcyt.2015.11.008 PMC4745114

[33] Bloom DD, Centanni JM, Bhatia N, Emler CA, Drier D, Leverson GE, et al. A reproducible immunopotency assay to measure mesenchymal stromal cell-mediated T-cell suppression. Cytotherapy. 2015;17(2):140–51. 25455739 10.1016/j.jcyt.2014.10.002 PMC4297551

[34] Galipeau J. Mesenchymal Stromal Cells: Clinical Challenges and Therapeutic Opportunities. Cell Stem Cell. 2018;22(6):824–33. 29859173 10.1016/j.stem.2018.05.004 PMC6434696

[35] Kaur G, Bae EH, Zhang Y, Ciacciofera N, Jung KM, Barreda H, et al. Biopotency and surrogate assays to validate the immunomodulatory potency of extracellular vesicles derived from mesenchymal stem/stromal cells for the treatment of experimental autoimmune uveitis. J Extracell Vesicles. 2024;13(8). 10.1002/jev2.12497 PMC11322862 39140452

[36] Liu H, Liu SY, Qiu XY, Yang XH, Bao LL, Pu FX, et al. Donor MSCs release apoptotic bodies to improve myocardial infarction via autophagy regulation in recipient cells. Autophagy. 2020;16(12):2140–55. 31959090 10.1080/15548627.2020.1717128 PMC7751634

[37] Jing Wen D, Creaven X, Luan J, Wang. Comparison of immunotherapy mediated by apoptotic bodies, microvesicles and exosomes: apoptotic bodies’ unique anti-inflammatory potential. J Transl Med. 2023;21(1):478. 37461033 10.1186/s12967-023-04342-w PMC10353199

[38] Jiemin Wang S, Moosavizadeh M, Jammes A, Tabasi T, Bach, Aideen E, Ryan, et al. Comparison of in-vitro immunomodulatory capacity between large and small apoptotic bodies from human bone marrow mesenchymal stromal cells. Int Immunopharmacol. 2025;153. 10.1016/j.intimp.2025.114480 40101418

[39] Ragni E, Orfei CP, De Luca P, Mondadori C, Vigano M, Colombini A, et al. Inflammatory priming enhances mesenchymal stromal cell secretome potential as a clinical product for regenerative medicine approaches through secreted factors and EV-miRNAs: the example of joint disease. Stem Cell Res Ther. 2020;11(1):19. 32345351 10.1186/s13287-020-01677-9 PMC7189600

[40] Riazifar M, Mohammadi MR, Pone EJ, Yeri A, Lasser C, Segaliny AI, et al. Stem Cell-Derived Exosomes as Nanotherapeutics for Autoimmune and Neurodegenerative Disorders. ACS Nano. 2019;13(6):6670–88. 31117376 10.1021/acsnano.9b01004 PMC6880946

[41] Gompf K, Traverso M. J Hetterich. Using Analytical Hierarchy Process (AHP) to introduce weights to social life cycle assessment of mobility services. Sustainability. 2021;13(3).

[42] Principal Components Analysis (PCA). Better Explained. Available from: https://www.machinelearningplus.com/machine-learning/principal-components-analysis-pca-better-explained/.

[43] Zhu YX, Tian DZ, Yan F. Effectiveness of entropy weight method in decision-making. Math Probl Eng. 2020;2020.

[44] Huling JD, Greifer N, Chen GH. Independence weights for causal inference with continuous treatments. J Am Stat Assoc. 2024;119:1657–70.

[45] He HP, Takahashi A, Mukai T, Hori A, Narita M, Tojo A, et al. The immunomodulatory effect of triptolide on mesenchymal stromal cells. Front Immunol. 2021;12. 10.3389/fimmu.2021.686356 PMC8415460 34484183

[46] Jiemin. Wang S, Moosavizadeh AE, Ryan, et al. In-vitro immunomodulatory efficacy of extracellular vesicles derived from TGF-beta1/IFN-gamma dual licensed human bone marrow mesenchymal stromal cells. Stem Cell Res Ther. 2025;16(1):357. 10.1186/s13287-025-04476-2 PMC12239388 40629458

[47] Ou Q, Cormican S, Power R, Hontz S, Hanley SA, Islam MN, et al. Initial or continuous coculture with umbilical cord-derived mesenchymal stromal cells facilitates in vitro expansion of human regulatory T-cell subpopulations. Stem Cells Transl Med. 2025;14(6). 10.1093/stcltm/szaf012 PMC12166524 40515654

[48] Salem B, Miner S, Hensel NF, Battiwalla M, Keyvanfar K, Stroncek DF, et al. Quantitative activation suppression assay to evaluate human bone marrow-derived mesenchymal stromal cell potency. Cytotherapy. 2015;17(12):1675–86. 26422657 10.1016/j.jcyt.2015.08.008 PMC4655179

[49] Myneni VD, McClain-Caldwell I, Martin D, Vitale-Cross L, Marko K, Firriolo JM, et al. Mesenchymal stromal cells from infants with simple polydactyly modulate immune responses more efficiently than adult mesenchymal stromal cells. Cytotherapy. 2019;21(2):148–61. 30595353 10.1016/j.jcyt.2018.11.008 PMC6435420

[50] Ou QF, Power R, Griffin MD. Revisiting regulatory T cells as modulators of innate immune response and inflammatory diseases. Front Immunol. 2023;14. 10.3389/fimmu.2023.1287465 PMC10623442 37928540

[51] Mora-García MD, Garcia-Rocha R, Morales-Ramírez O, Montesinos JJ, Weiss-Steider B, Hernández-Montes J, et al. Mesenchymal stromal cells derived from cervical cancer produce high amounts of adenosine to suppress cytotoxic T lymphocyte functions. J Transl Med. 2016;14. 10.1186/s12967-016-1057-8 PMC5080842 27782859

[52] Chen XT, Shao H, Zhi YT, Xiao Q, Su C, Dong LJ, et al. CD73 Pathway Contributes to the Immunosuppressive Ability of Mesenchymal Stem Cells in Intraocular Autoimmune Responses. Stem Cells Dev. 2016;25(4):337–46. 26650818 10.1089/scd.2015.0227

[53] Gonzalez YIR, Lynch PJ, Thompson EE, Stultz BG. Hursh. In vitro cytokine licensing induces persistent permissive at the Indoleamine 2,3-dioxygenase promoter. Cytotherapy. 2016;18(9):1114–28. 27421739 10.1016/j.jcyt.2016.05.017 PMC4983509

[54] Mazzoni A, Maggi L, Montaini G, Ramazzotti M, Capone M, Vanni A, et al. Human T cells interacting with HNSCC-derived mesenchymal stromal cells acquire tissue-resident memory like properties. Eur J Immunol. 2020;50(10):1571–9. 32441311 10.1002/eji.202048544

[55] Selich A, Zimmermann K, Tenspolde M, Dittrich-Breiholz O, von Kaisenberg C, Schambach A, et al. Umbilical cord as a long-term source of activatable mesenchymal stromal cells for immunomodulation. Stem Cell Res Ther. 2019;10(1). 10.1186/s13287-019-1376-9 PMC6755709 31547865

[56] Diedrichs F, Stolk M, Jürchott K, Haag M, Sittinger M, Seifert M. Enhanced immunomodulation in inflammatory environments favors human cardiac mesenchymal stromal-like cells for allogeneic cell therapies. Front Immunol. 2019;10. 10.3389/fimmu.2019.01716 PMC6665953 31396228

[57] Fang XH, Abbott J, Cheng LD, Colby JK, Lee JW, Levy BD, et al. Human Mesenchymal Stem (Stromal) Cells Promote the Resolution of Acute Lung Injury in Part through Lipoxin A4. J Immunol. 2015;195(3):875–81. 26116507 10.4049/jimmunol.1500244

[58] Deng JQ, Li DT, Huang XY, Li WY, Zhao FF, Gu CW, et al. Interferon-γ enhances the immunosuppressive ability of canine bone marrow-derived mesenchymal stem cells by activating the TLR3-dependent IDO/kynurenine pathway. Mol Biol Rep. 2022;49(9):8337–47. 35690960 10.1007/s11033-022-07648-y

[59] Herzig MC, Christy BA, Montgomery RK, Delavan CP, Jensen KJ, Lovelace SE, et al. Interactions of human mesenchymal stromal cells with peripheral blood mononuclear cells in a mitogenic proliferation assay. J Immunol Methods. 2021;492. 10.1016/j.jim.2021.113000 33609532

[60] Bai XL, Chen TW, Li YQ, Ge XF, Qiu CE, Gou HL, et al. PD-L1 expression levels in mesenchymal stromal cells predict their therapeutic values for autoimmune hepatitis. Stem Cell Res Ther. 2023;14(1). 10.1186/s13287-023-03594-z PMC10729378 38111045

[61] Fattore AD, Luciano R, Pascucci L, Goffredo BM, Giorda E, Scapaticci M, et al. Immunoregulatory Effects of Mesenchymal Stem Cell-Derived Extracellular Vesicles on T Lymphocytes. Cell Transplant. 2015;24(12):2615–27. 25695896 10.3727/096368915X687543

[62] Han D, Li Z, Wang F, Cheng K, Shen D. Apoptotic extracellular vesicles: mechanisms, applications, and therapeutic potential. Med-X. 2024;2(1):27.

[63] Yu L, Zhu G, Zhang Z, Yu Y, Zeng L, Xu Z, et al. Apoptotic bodies: bioactive treasure left behind by the dying cells with robust diagnostic and therapeutic application potentials. J Nanobiotechnol. 2023;21(1):218. 10.1186/s12951-023-01969-1 PMC10337089 37434199

[64] Zernecke A, Bidzhekov K, Noels H, Shagdarsuren E, Gan L, Denecke B, et al. Delivery of microRNA-126 by apoptotic bodies induces CXCL12-dependent vascular protection. Sci Signal. 2009;2(100):ra81. 19996457 10.1126/scisignal.2000610

[65] Nguyen DB, Ly TB, Wesseling MC, Hittinger M, Torge A, Devitt A, et al. Characterization of Microvesicles Released from Human Red Blood Cells. Cell Physiol Biochem. 2016;38(3):1085–99. 26938586 10.1159/000443059

[66] Xu X, Lai Y, Hua ZC. Apoptosis and apoptotic body: disease message and therapeutic target potentials. Biosci Rep. 2019;39(1). 10.1042/BSR20180992 PMC6340950 30530866

[67] Battistelli M, Falcieri E. Apoptotic bodies: particular extracellular vesicles involved in intercellular communication. Biology (Basel). 2020;9(1). 10.3390/biology9010021 PMC7168913 31968627

[68] Stagg J, Pommey S, Eliopoulos N, Galipeau J. Interferon-gamma-stimulated marrow stromal cells: a new type of nonhematopoietic antigen-presenting cell. Blood. 2006;107(6):2570–7. 16293599 10.1182/blood-2005-07-2793

[69] Chan WK, Lau AS, Li JC, Law HK, Lau YL. Chan. MHC expression kinetics and immunogenicity of mesenchymal stromal cells after short-term IFN-gamma challenge. Exp Hematol. 2008;36(11):1545–55. 18715686 10.1016/j.exphem.2008.06.008

[70] Chinnadurai R, Copland IB, Patel SR, Galipeau J. IDO-independent suppression of T cell effector function by IFN-gamma-licensed human mesenchymal stromal cells. J Immunol. 2014;192(4):1491–501. 24403533 10.4049/jimmunol.1301828

[71] Shadmanfar S, Labibzadeh N, Emadedin M, Jaroughi N, Azimian V, Mardpour S, et al. Intra-articular knee implantation of autologous bone marrow-derived mesenchymal stromal cells in rheumatoid arthritis patients with knee involvement: Results of a randomized, triple-blind, placebo-controlled phase 1/2 clinical trial. Cytotherapy. 2018;20(4):499–506. 29428486 10.1016/j.jcyt.2017.12.009

[72] Liu J, Qiu X, Lv Y, Zheng C, Dong Y, Dou G, et al. Apoptotic bodies derived from mesenchymal stem cells promote cutaneous wound healing via regulating the functions of macrophages. Stem Cell Res Ther. 2020;11(1):507. 33246491 10.1186/s13287-020-02014-w PMC7694913

[73] Donohoe E, Canning A, Johnston E, Moosavizadeh S, Wang J, Leahy M, et al. Small extracellular vesicles secreted from TGF-beta1-licensed mesenchymal stromal cells reduce inflammation-associated injury following corneal alkali burn. Stem Cell Res Ther. 2025;16(1):376. 40660344 10.1186/s13287-025-04504-1 PMC12261806

[74] Meenakshi Sundaram R, Kadapakkam Nandabalan S, Rupert S, Srinivasan P, Sankar P, Patra B, et al. Differential immunomodulation of human mesenchymal stromal cells from various sources in an inflammation mimetic milieu. Cytotherapy. 2022;24(2):110–23. 34740526 10.1016/j.jcyt.2021.09.005

[75] Mattar P, Bieback K. Comparing the immunomodulatory properties of bone marrow, adipose tissue, and birth-associated tissue mesenchymal stromal cells. Front Immunol. 2015;6. 10.3389/fimmu.2015.00560 PMC4630659 26579133

[76] Arki MK, Moeinabadi-Bidgoli K, Niknam B, Mohammadi P, Hassan M, Hossein-Khannazer N, et al. Immunomodulatory performance of GMP-compliant, clinical-grade mesenchymal stromal cells from four different sources. Heliyon. 2024;10(2):e24948. 38312681 10.1016/j.heliyon.2024.e24948 PMC10835001

