# Supplementary material for: Quantitative evaluation of mesenchymal stromal cell immunomodulatory potency and cost-effectiveness of cytokine licensing for translational application
Source: J Transl Med. 2026 Mar 4;24:496. doi: 10.1186/s12967-026-07947-z (PMC13069805; doi:10.1186/s12967-026-07947-z)
Supplement: Supplementary file 1 — Supplementary Material 1 [file 12967_2026_7947_MOESM1_ESM.docx]

1. **R Script for Calculating the Weight**

***By Entropy***

**# **Load necessary packages****

library(dplyr)

library(tidyr)

**# **Read the data****

file_path <- "Cytokine_potency_table_Aran_3_matrix.csv"

data <- read.csv(file_path)

**# **Extract numerical columns (excluding the Group column)****

value_cols <- setdiff(colnames(data), "Group")

**# **1. Data standardization (Z-Score)****

data_zscore <- data %>%

mutate(across(all_of(value_cols), ~ (. - mean(. , na.rm = TRUE)) / sd(., na.rm = TRUE)))

**# **2. Entropy Weight Method Calculation****

# **(a) Transform values to positive to avoid log(0) issues**

data_positive <- data_zscore %>%

mutate(across(all_of(value_cols), ~ . - min(., na.rm = TRUE) + 1e-6))

# **(b) Calculate the proportion of each value p_ij**

p <- data_positive %>%

mutate(across(all_of(value_cols), ~ ./sum(., na.rm = TRUE)))

# **(c) Calculate entropy**

k <- 1 / log(nrow(p)) # **Constant k**

entropy <- p %>%

summarise(across(all_of(value_cols), ~ -k * sum(ifelse(. > 0, . * log(.), 0), na.rm = TRUE)))

# **(d) Calculate redundancy d_j**

redundancy <- 1 - entropy

# **(e) Calculate weights w_j**

weights <- redundancy / sum(redundancy)

# **Convert weights to a data frame**

weights_df <- data.frame(Indicator = value_cols, Weight = as.numeric(weights))

**# **3. Merge weights with standardized data****

# **Save the weight table and standardized data separately to avoid conflicts**

output_path_data <- "Cytokine_potency_standardized_data55.csv"

output_path_weights <- "Cytokine_potency_weights55.csv"

write.csv(data_zscore, output_path_data, row.names = FALSE)

write.csv(weights_df, output_path_weights, row.names = FALSE)

**# **Print output path prompts****

cat("Standardized data saved to: ", output_path_data, "\n")

cat("Weight table saved to: ", output_path_weights, "\n")

***By Analytic hierarchy process***

**# **Load necessary libraries****

library(readr)

**# **Set file paths****

ahp_file <- "AHP_point_3.csv"

output_weights_file <- "indicator_weight_4.csv"

**# **Load AHP data****

ahp_data <- read_csv(ahp_file)

**# **Extract the matrix portion****

ahp_matrix <- as.matrix(ahp_data[, -1])

**# **Function to enforce reciprocal matrix property****

fix_reciprocal_matrix <- function(matrix) {

n <- nrow(matrix)

for (i in 1:n) {

for (j in 1:n) {

if (i != j) {

# **Forcefully set a_ji = 1 / a_ij**

matrix[j, i] <- 1 / matrix[i, j]

}

}

}

return(matrix)

}

**# **Fix the reciprocal matrix****

ahp_matrix_fixed <- fix_reciprocal_matrix(ahp_matrix)

**# **Recheck the fixed matrix****

tolerance <- 1e-6 # **Error tolerance**

reciprocal_check_fixed <- all(abs(ahp_matrix_fixed * t(1 / ahp_matrix_fixed) - 1) < tolerance, na.rm = TRUE)

if (!reciprocal_check_fixed) {

stop("The fixed scoring matrix still has issues. Please check the data!")

} else {

cat("The fixed scoring matrix has passed the reciprocity check.\n")

}

**# **Proceed to weight calculation****

**# **Compute eigenvalues and eigenvectors****

eigen_analysis <- eigen(ahp_matrix_fixed)

weights <- Re(eigen_analysis$vectors[, 1]) # **Extract the real part**

weights <- weights / sum(weights) # **Normalize**

**# **Create the weight table****

indicator_weights <- data.frame(

Indicator = ahp_data$Indicator,

Weight = weights

)

**# **Save the weight table****

write_csv(indicator_weights, output_weights_file)

cat("The weight table has been saved to:", output_weights_file, "\n")

***By Independence***

**# **Load necessary packages****

library(dplyr)

library(tidyr)

**# **Read the data****

data <- read.csv("Cytokine_potency_table_Aran_3_average.csv")

**# **Step 1: Perform Z-standardization by Indicator****

data <- data %>%

group_by(Indicator) %>%

mutate(Z_Score = (Value - mean(Value, na.rm = TRUE)) / sd(Value, na.rm = TRUE)) %>%

ungroup()

**# **Step 2: Create a matrix for correlation calculation****

pivot_data <- data %>%

select(Group, Indicator, Z_Score) %>%

pivot_wider(names_from = Indicator, values_from = Z_Score)

**# **Set Group as row names****

pivot_data <- as.data.frame(pivot_data)

rownames(pivot_data) <- pivot_data$Group

pivot_data$Group <- NULL

**# **Ensure all columns are numeric****

pivot_data <- pivot_data %>%

mutate(across(everything(), as.numeric))

**# **Step 3: Calculate the correlation matrix****

correlation_matrix <- cor(pivot_data, use = "pairwise.complete.obs")

**# **Compute independence weights (1 - mean of |correlation|)****

independence_weights <- apply(correlation_matrix, 1, function(row) {

mean(1 - abs(row), na.rm = TRUE)

})

**# **Normalize weights so their sum equals 1****

normalized_weights <- independence_weights / sum(independence_weights)

**# **Step 4: Append weights to the original data****

data <- data %>%

mutate(Weight = normalized_weights[match(Indicator, names(normalized_weights))])

**# **Save the results to the default directory****

write.csv(data, "Cytokine_potency_table_with_Weights.csv", row.names = FALSE)

**# **Output completion message****

cat("Results have been saved as 'Cytokine_potency_table_with_Weights.csv'")

***By Principal component analysis***

**#** Load necessary libraries**#**

library(factoextra)

**#** Step 1: Load the dataset**

**# **Replace with your file path**

data <- read.csv("Cytokine_potency_table_Aran_4_matrix_PCA.csv", row.names = 1)

**# **Step 2: Standardize the dataset column-wise (Z-score normalization)**

data_scaled <- scale(data, center = TRUE, scale = TRUE)

**#** Step 3: Perform PCA**

pca_result <- prcomp(data_scaled, center = TRUE, scale. = TRUE)

**#** Step 4: Calculate variable contributions (weights) from PCA**

**# **Get the proportion of variance explained by each principal component**

explained_variance <- pca_result$sdev^2 / sum(pca_result$sdev^2)

**#** Get the variable contributions to the principal components**

loadings <- pca_result$rotation # PCA loadings (eigenvectors)

variable_contributions <- abs(loadings) %*% explained_variance # Weighted contributions

**# **Normalize contributions to sum to 1 (optional, depending on use case)**

variable_weights <- variable_contributions / sum(variable_contributions)

**# **Step 5: Create a data frame for the results**

weights_df <- data.frame(

Variable = rownames(loadings),

Weight = as.vector(variable_weights)

)

**#** Step 6: Save results to a CSV file (optional)**

write.csv(weights_df, "PCA_Variable_Weights.csv", row.names = FALSE)

**#** Print the weights**

print(weights_df)
