# Supplementary material for: Quantitative evaluation of mesenchymal stromal cell immunomodulatory potency and cost-effectiveness of cytokine licensing for translational application
Source: J Transl Med. 2026 Mar 4;24:496. doi: 10.1186/s12967-026-07947-z (PMC13069805; doi:10.1186/s12967-026-07947-z)
Supplement: Supplementary file 3 — Supplementary Material 3 [file 12967_2026_7947_MOESM3_ESM.docx]

1. ***Estimating AHP Weights***

| λ_max_ (Principal Eigenvalue) | Consistency Index (CI) | Random Consistency Index (RI) | Consistency Ratio (CR) |
| --- | --- | --- | --- |
| 16.16488 | 0.08320573 | 1.49 | 0.05584277 |

$$Consistency Ratio (CR)=\frac{Consistency Index (CI)}{Random Consistency Index (RI)}$$

In an ideal scenario, a perfectly consistent judgment matrix satisfies λmax=n (where n is the matrix dimension). In our case, the matrix dimension is n=15, while λmax=16.16488, which is slightly larger than 15. This indicates a minor inconsistency in the judgment matrix.

The Consistency Index (CI) measures the deviation of the judgment matrix's consistency, with values closer to 0 indicating better consistency. Our results show a slight deviation in CI, but it is not significant.

The Consistency Ratio (CR) assesses whether the judgment matrix's consistency falls within an acceptable range. Typically, a CR value less than 0.1 is considered acceptable. In our case, CR=0.0558, which meets the consistency requirement, confirming that the matrix's consistency is acceptable.

1. ***Highly Relevant Indicators***

| Variable 1 | Variable 2 | Correlation |
| --- | --- | --- |
| C1s RNA Expression | C1s Protein Concentration | 0.965474 |
| C1s RNA Expression | IDO1 RNA Expression | 0.936103 |
| C1s RNA Expression | IDO1 Protein Expression | 0.839939 |
| C1s RNA Expression | MX2 RNA Expression | 0.800508 |
| C1s RNA Expression | MX2 Protein Expression | 0.833958 |
| C1s RNA Expression | PD-L1 RNA Expression | 0.832789 |
| C1s Protein Concentration | IDO1 RNA Expression | 0.880326 |
| C1s Protein Concentration | MX2 mRNA Expression | 0.860582 |
| C1s Protein Concentration | MX2 Protein Expression | 0.819132 |
| CD3+ T Cell Proliferation | CD4+ T Cell Proliferation | 0.98939 |
| CD3+ T Cell Proliferation | CD8+ T Cell Proliferation | 0.966584 |
| CD4+ T Cell Proliferation | CD8+ T Cell Proliferation | 0.968081 |
| CD73 Expression Level | CD73 Positivity Rate | 0.896898 |
| IDO1 RNA Expression | IDO1 Protein Expression | 0.944431 |
| IDO1 RNA Expression | PD-L1 RNA Expression | 0.845322 |
| IDO1 Protein Expression | PD-L1 RNA Expression | 0.860074 |
| Kynurenine (KYN) Concentration | PD-L1 RNA Expression | 0.942081 |
| Kynurenine (KYN) Concentration | PD-L1 Protein Expression | 0.843744 |
| PD-L1 mRNA Expression | PD-L1 Protein Expression | 0.8349 |

1. ***Estimating PCA Weights***


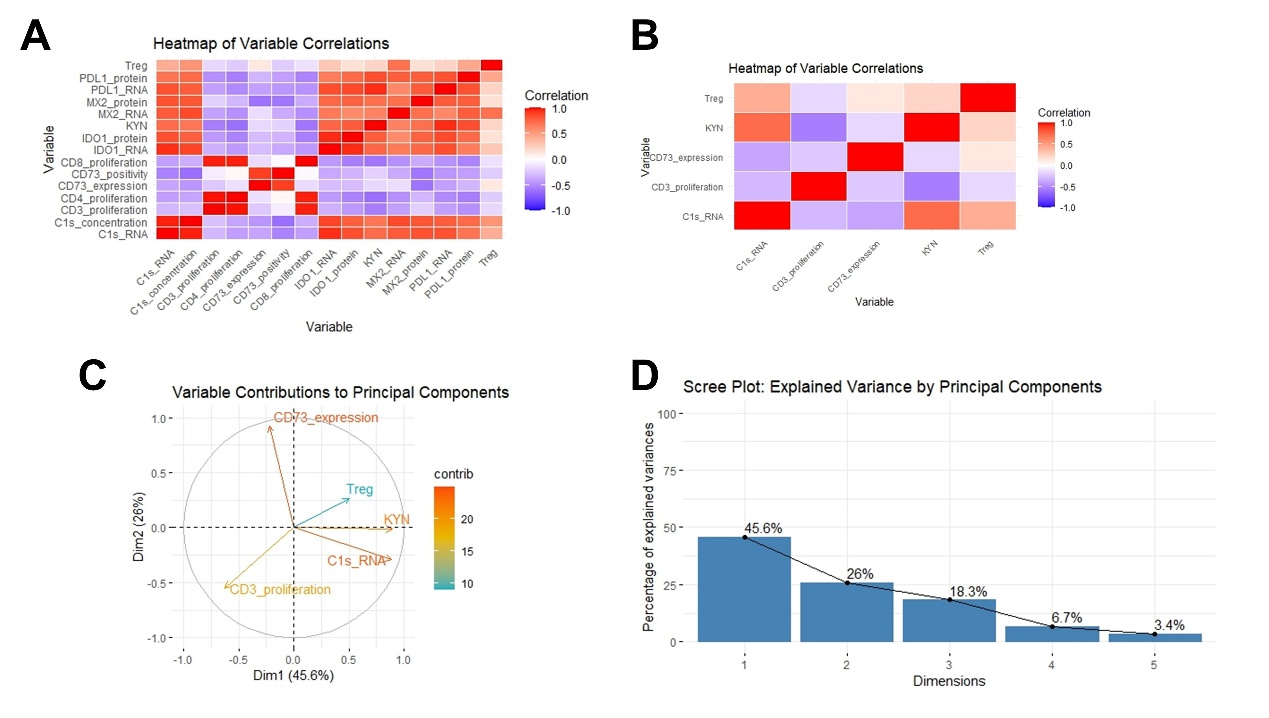


**Supplementary Material Figure 1.**
A. Heatmap of indicator correlations.
B. Heatmap of indicator correlations after removing highly relevant indicators.
C. Variable (indicator) contributions to principal components.
D. Explained variance by principal components.
